# Correlation of Population SARS-CoV-2 Cycle Threshold Values to Local Disease Dynamics: Exploratory Observational Study

**DOI:** 10.2196/28265

**Published:** 2021-06-03

**Authors:** Chak Foon Tso, Anurag Garikipati, Abigail Green-Saxena, Qingqing Mao, Ritankar Das

**Affiliations:** 1 Dascena, Inc Houston, TX United States

**Keywords:** reverse transcription polymerase chain reaction, testing, cycle threshold, COVID-19, epidemiology, Rt, exploratory, correlation, population, threshold, disease dynamic, distribution, transmission

## Abstract

**Background:**

Despite the limitations in the use of cycle threshold (CT) values for individual patient care, population distributions of CT values may be useful indicators of local outbreaks.

**Objective:**

We aimed to conduct an exploratory analysis of potential correlations between the population distribution of cycle threshold (CT) values and COVID-19 dynamics, which were operationalized as percent positivity, transmission rate (R_t_), and COVID-19 hospitalization count.

**Methods:**

In total, 148,410 specimens collected between September 15, 2020, and January 11, 2021, from the greater El Paso area were processed in the Dascena COVID-19 Laboratory. The daily median CT value, daily R_t_, daily count of COVID-19 hospitalizations, daily change in percent positivity, and rolling averages of these features were plotted over time. Two-way scatterplots and linear regression were used to evaluate possible associations between daily median CT values and outbreak measures. Cross-correlation plots were used to determine whether a time delay existed between changes in daily median CT values and measures of community disease dynamics.

**Results:**

Daily median CT values negatively correlated with the daily R_t_ values (*P*<.001), the daily COVID-19 hospitalization counts (with a 33-day time delay; *P*<.001), and the daily changes in percent positivity among testing samples (*P*<.001). Despite visual trends suggesting time delays in the plots for median CT values and outbreak measures, a statistically significant delay was only detected between changes in median CT values and COVID-19 hospitalization counts (*P*<.001).

**Conclusions:**

This study adds to the literature by analyzing samples collected from an entire geographical area and contextualizing the results with other research investigating population CT values.

## Introduction

As of February 16, 2021, the SARS-CoV-2 virus has infected more than 109 million people around the world and has been implicated in 2.41 million deaths [1]. In the United States alone, more than 486,500 deaths have been attributed to COVID-19 [1]. Reverse transcription polymerase chain reaction (RT-PCR) testing has become the predominant method for COVID-19 surveillance and diagnostic testing due to having higher sensitivity, higher specificity, and faster turnaround times compared to those of viral cultures [2,3]. RT-PCR tests detect viral genetic material in biological samples [4]. The cycle threshold (CT) value represents the number of polymerase chain reaction cycles that are required to detect a positive signal [5]. The CT value is inversely related to the viral load; a 3.3 increase in CT value indicates an approximately 10-fold decrease in the amount of viral genetic material present in a sample [5]. COVID-19 RT-PCR tests are generally considered positive only if they generate a result with a CT value that is lower than the recommended cutoff. In the United States, the Food and Drug Administration has approved emergency use authorizations for tests in which CT values of <37 can be considered positive [6].

CT values are lowest—indicating a larger amount of viral genetic material—early in the disease course. Indeed, numerous studies have reported that CT values tend to be highest prior to or during the earliest days following the onset of symptoms and decline as a disease progresses [7-11]. Lower CT values have been directly linked with higher infectivity, as shown by researchers’ ability to cultivate live SARS-CoV-2 from samples [7,11-14] and the number of individuals infected by an index case [15]. In one study, SARS-CoV-2 could be cultivated from over 70% of samples with a CT value of <25 but could only be cultivated from less than 3% of samples with a CT value of ≥35 [16]. Singanayagam et al [11] found that in samples with a CT value of >35, the probability of such samples containing cultivable viruses declines to approximately 8%, and the correlations between CT values and the probability of samples containing cultivable viruses were similar in samples collected from symptomatic and asymptomatic individuals. CT values have also been reported to directly correlate with disease severity and mortality; CT values tend to be lower in patients with more severe disease presentations [13,17-19] and in hospitalized patients who ultimately die from COVID-19 [13,20].

There are however considerable limitations in the use of CT values for prognostication and treatment planning at the level of individual patients. Critics have noted that there may be significant variability in CT values based on the quantity of biological material collected on a testing swab as well as differences in RT-PCR reagents, equipment, and standards among laboratories [21]. CT values may also vary based on the gene target selected for RT-PCR or even based on the assay used to detect the same gene target [22]. In addition, RT-PCR only detects the presence of viral material and is unable to distinguish between live viruses and viral debris, which may linger for an extended period once an individual is no longer infectious [17]. CT values, when used as a semiquantitative measure of how much viral nucleic acid is present, are similarly limited. As a result of these constraints, clinicians and researchers continue to debate the utility of CT values for informing health care choices for individuals [5,21,22].

Despite the limitations in the use of individual-level CT values, measures of CT values across a population may provide a useful measure of COVID-19 dynamics in communities. It has been suggested that big data technology could be applied to the large amounts of data resulting from the pandemic in order to provide timely information for policy development [23]. As CT values have been reported to correlate with disease stage and infectivity, a higher proportion of low CT values in testing samples from a population may reflect epidemic growth in a community [24]. Preliminary analyses of simulation and surveillance testing data have suggested that decreases in the distribution of CT values in a population, as measured by the median CT value, may precede a local increase in transmission or positivity rates [24,25]. As such, the median CT value may be a useful measure for predicting a pandemic surge. This study describes an exploratory analysis of potential correlations between median CT values and COVID-19 dynamics, which were operationalized as percent positivity, transmission rate (R_t_), and COVID-19 hospitalization count.

## Methods

### Sample Selection

The samples included in this study were collected between September 15, 2020, and January 11, 2021, as part of the ongoing diagnostic evaluation services provided by Dascena, Inc to residents in the state of Texas. In the greater El Paso area, a contractor for the El Paso Department of Public Health sent over 90% of collected samples to the Dascena COVID-19 Laboratory in Houston, Texas. All samples from the greater El Paso area that were processed by Dascena, Inc during the study period were included in our analysis. Supplementary analyses also included samples from Houston-Sugarland-Baytown, Dallas-Fort Worth-Arlington, and Austin-Round Rock. The Pearl Independent Institutional Review Board (IRB) approved this study with a waiver of informed consent (IRB Protocol 21-DASC-127).

This study included nasopharyngeal swabs, salivary samples, an anterior nares swab sample, and samples for which the type of biological specimen was not specified. The overwhelming majority of samples (147,720/148,410, 99.54%) were nasopharyngeal swabs. All biological samples were sent to the Clinical Laboratory Improvement Amendments–certified Dascena Laboratory. All samples were analyzed with the TaqPath COVID-19 Combi Kit (Thermo Fisher Scientific), and extraction was performed with a MagMAX RNA Isolation Kit (Thermo Fisher Scientific). The following three gene targets are used by these assays and may be the source of a positive result: the nucleocapsid gene, the spike gene, and the *ORF1ab* (open reading frames 1ab) gene [26]. RT-PCR was only conducted once for any unique sample. For each RT-PCR test, the CT value was recorded. Only samples that produced a valid CT value for a positive COVID-19 test (ie, at least 2 genes generating a positive signal with a CT value of ≤37) were used to determine daily median CT values and used in subsequent correlation analyses.

### Data Processing and Measures

The following demographic data were available for testing samples: age, sex, race, ethnicity, and zip codes of residences. Testing samples from the greater El Paso area were selected based on the zip codes that were listed as part of the El Paso metropolitan statistical area (MSA) by the US Department of Labor, Office of Workers Compensation Program [27]. Daily percent positivity rate was calculated among all of the samples tested by Dascena from the greater El Paso area.

The effective reproduction number or R_t_ value was derived using the open-source algorithm from the rtcovidlive COVID-19 tracking website [28]. The algorithm is a Python script based on a Bayesian estimation model developed by Bettencourt and Ribeiro [29] with slight modifications for introducing gaussian noise to the prediction. Daily, new COVID-19 case data from individual counties were obtained from the COVID-19 Dashboard of the Center for Systems Science and Engineering at Johns Hopkins University [1], grouped by MSA, and fed into the rtcovidlive algorithm to generate a time series for R_t_. The daily number of hospitalized individuals with COVID-19 in the El Paso area was derived from publicly available data produced by the Texas Department of State Health Services, which are grouped by trauma service area [30].

### Comparative Analysis of Population-Level CT Values

In order to contextualize the results, a focused literature review using title and keyword searches was performed for peer-reviewed publications and preprint manuscripts on the use of CT value measurements across a population as a means of predicting or monitoring COVID-19 outbreaks. In total, 3 preprints were identified [24,25,31]. The data sets from this study and the preprints were then compared in terms of source population, the type of testing, sample size, the biological sample types included, the duration of the study period, the gene target(s) of RT-PCR tests, the CT-based value(s) measured, the metrics used to measure COVID-19 outbreaks, and the outcomes of the study.

### Statistical Analysis

All analyses were conducted in Python [32] by using the following packages: pandas, matplotlib, plotly, scipy, and statsmodels. The daily median CT value among Dascena test samples, the daily R_t_ in the El Paso MSA, and the daily count of hospitalized individuals with COVID-19 in El Paso were plotted over time. Rolling 7-day averages of daily median CT values (with a minimum 5 days of data present in the window), the daily R_t_, the daily number of COVID-19 hospitalizations, and the daily percent positivity rate among samples from El Paso that were sent to the Dascena Laboratory were also plotted over time. To better capture the dynamic change in percent positivity among Dascena test samples, the daily change in percent positivity was calculated from the 7-day rolling average for days in which more than 200 total tests were performed by the Dascena Laboratory. If fewer than 200 tests were performed on a particular day (eg, due to the holiday shutdown of collection sites), the percent positivity from the previous day was carried forward. The daily change in percent positivity was then plotted over time.

Scatterplots and linear regression were used to evaluate possible associations between the daily median CT value (nucleocapsid gene) and daily R_t_, between the daily median CT value (nucleocapsid gene) and the daily count of COVID-19 hospitalizations, and between the daily median CT value (nucleocapsid gene) and the daily change in percent positivity among samples processed by Dascena. Since a considerable time delay was observed between changes in the daily median CT value (nucleocapsid gene) and the daily count of COVID-19 hospitalizations, a time lag of 33 days was applied to the hospitalization data prior to creating the scatterplot and conducting linear regression. Median CT values based on the nucleocapsid gene were selected because they have previously been cited in research on population CT values [24,30].

In order to evaluate whether a time delay existed between changes in the daily median CT value (nucleocapsid gene) and community outbreaks, cross-correlation plots were constructed between the daily median CT value and daily R_t_, between the daily median CT value and the daily count of hospitalized patients with COVID-19, and between the daily median CT value and the daily change in percent positivity. In brief, a cross-correlation coefficient was obtained by dividing the correlation between two signals by the product of the auto-correlation of each of the two signals. The argmax of the cross-correlation coefficient is the dominant lag time between the two signals. As the purpose of our analysis was to investigate how the trough of daily median CT values correlated with the peak of the other signals, the following modifications were made to aid with visualization: (1) for each signal, the z-score was used instead of the absolute value; (2) the negative value of the z-score of the daily median CT value was used to ensure that a positive peak in the cross-correlation plots was present; and (3) 20% of positive samples were randomly sampled 5 times each day to estimate the variation in the cross-correlation between the daily median CT value and epidemiological signals. A 1-sample, two-tailed *t* test was used to determine if the mean lag differed statistically significantly from the 0.

Pairwise comparisons were performed via Pearson correlation (significance level of *P*<.05) to determine if any demographic factors that were associated with testing samples were significantly associated with R_t_, COVID-19 hospitalization count, or percent positivity. The following demographic factors were investigated: the daily number of tests, daily median age, the daily percentage of samples from men, the daily percentage of samples from individuals who indicated that they were White, and the daily percentage of samples from individuals who indicated that they were of Hispanic ethnicity.

## Results

In the greater El Paso area, 148,410 COVID-19 tests were sent to the Dascena Laboratory for processing, and 36,306 tests were positive. Of the 148,410 samples, 147,720 (99.54%) samples were nasopharyngeal swabs, 28 (0.02%) were salivary samples, 1 sample (0%) was an anterior nares swab, and 661 samples were biological specimens (0.45%) for which the type of specimen was not recorded. The median CT value (nucleocapsid gene) for nasopharyngeal samples was 23.14, which differed significantly from the median CT value (nucleocapsid gene; 25.58) observed for all other sample types (*P*<.001; Mood median test). The demographic characteristics of the entire population who were tested for COVID-19 are presented in Table 1.

**Figure 1:**
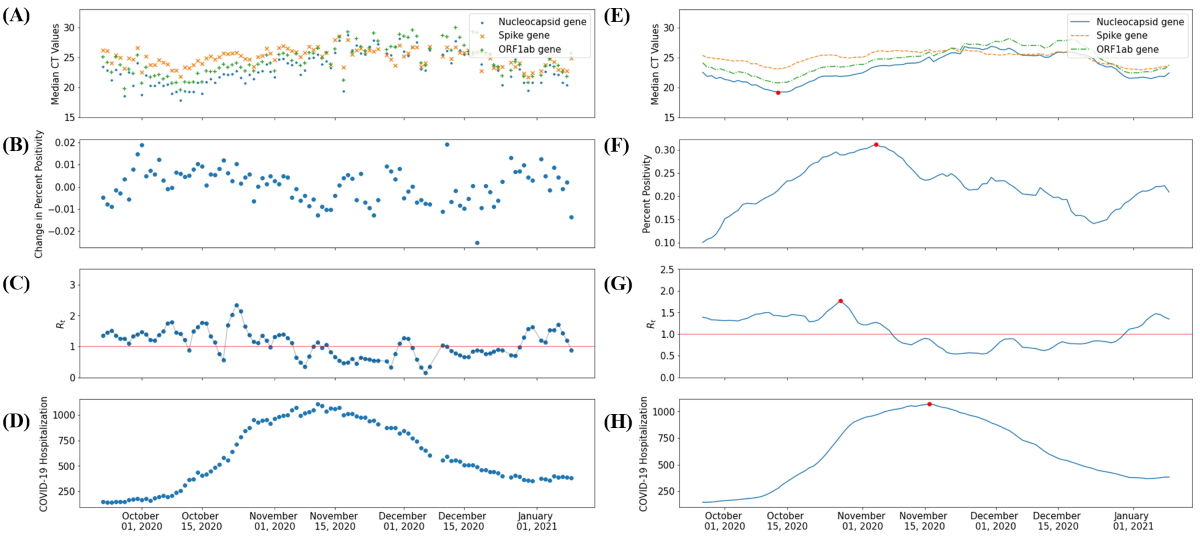
(A) Daily median CT values for SARS-CoV-2 positive samples. (B) Daily change in percent positivity for SARS-CoV-2 detection. (C) Daily SARS-CoV-2 R_t_. (D) Daily count of hospitalized individuals with COVID-19. (E) The 7-day rolling average of the daily median CT values for SARS-CoV-2–positive samples. (F) The 7-day rolling average of percent positivity rates for SARS-CoV-2–positive samples. (G) The 7-day rolling average of daily SARS-CoV-2 R_t_ values. (H) The 7-day rolling average of the number of hospitalized individuals with COVID-19 in the greater El Paso area between September 15, 2020, and January 11, 2021. The red lines in graphs C and G signify an R_t_ value of 1. Red dots represent the global minimum for smoothed CT values and global maxima for smoothed epidemiological indicators. CT: cycle threshold; *ORF1ab*: open reading frames 1ab; R_t_: transmission rate.

**Figure 2:**
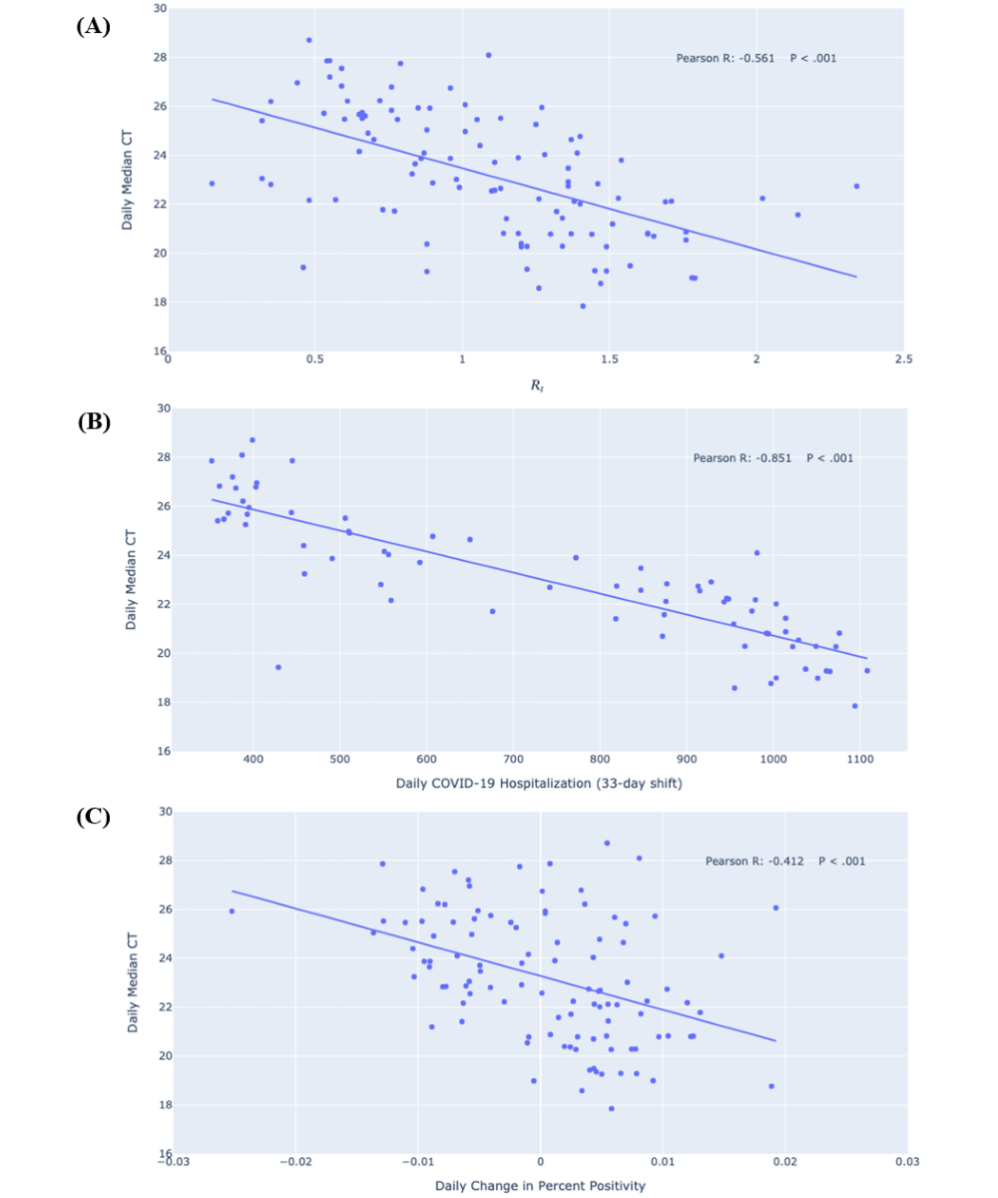
Linear regressions and scatter plots of (A) daily median CT values for SARS-CoV-2–positive samples versus daily transmission rate SARS-CoV-2 R_t_, (B) daily numbers of hospitalized individuals with COVID-19, and (C) daily changes in percent positivity for SARS-CoV-2 detection in the greater El Paso area between September 15, 2020, and January 11, 2021. CT: cycle threshold; R_t_: transmission rate.

Variability over time was observed in the median CT values and measures of COVID-19 dynamics in El Paso (Figure 1). As predicted in the a priori hypothesis, the daily median CT value negatively correlated with the daily R_t_, daily count of COVID-19 hospitalization (with a time delay), and daily change in percent positivity among testing samples in the greater El Paso area (Figure 2).

**Figure 3:**
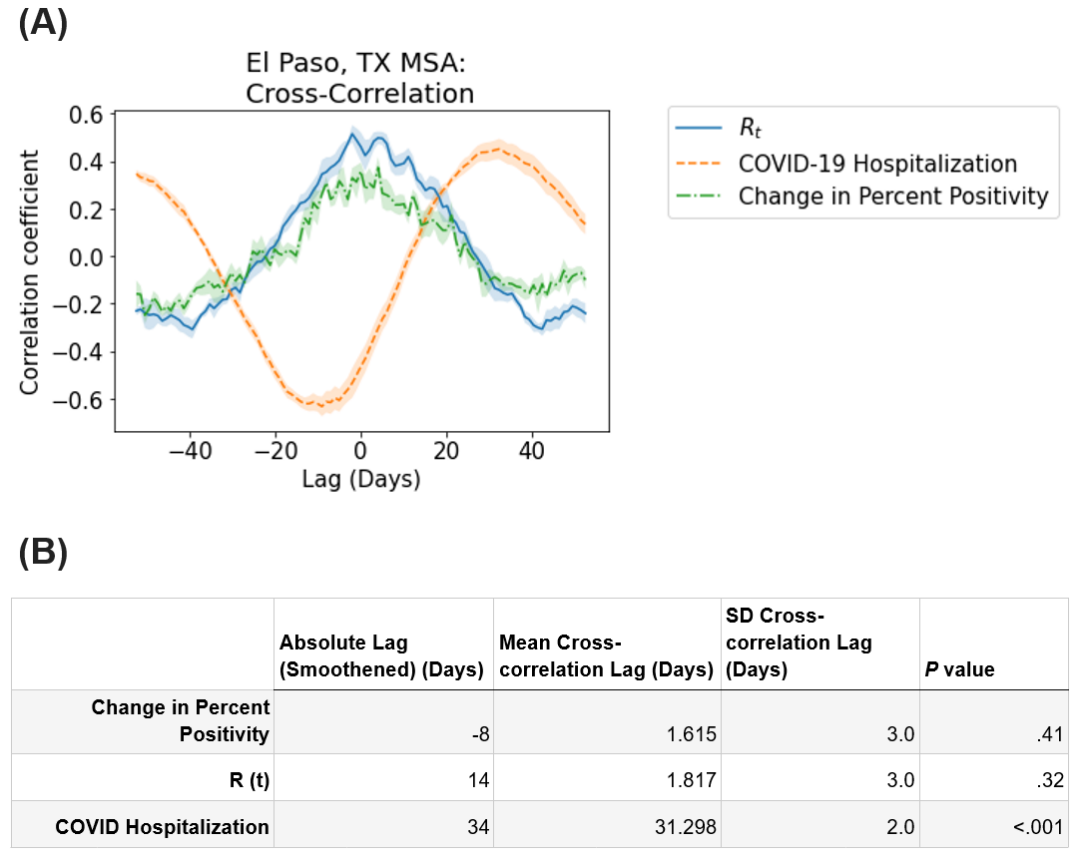
(A) Cross-correlation plot of daily median CT values for SARS-CoV-2–positive samples versus the daily SARS-CoV-2 R_t_, daily number of hospitalized individuals with COVID-19, and daily change in percent positivity for SARS-CoV-2 detection in the greater El Paso area between September 15, 2020, and January 11, 2021. Lines represent the mean, while shaded areas indicate the SD across 5-fold sampling. (B) Lag between COVID-19 epidemiological signals and the daily median CT (nucleocapsid gene) values for SARS-CoV-2–positive samples. Absolute lag (smoothened) is the absolute time difference between the peak of each epidemiological signal and the trough of daily median CT values with a 7-day rolling average (red dots in Figure 1B). Mean cross-correlation lag and SD cross-correlation lag represent the mean and SD, respectively, among lags determined by the 5-fold sampling of daily median CT values and cross-correlation. The *P* value column shows the *P* values for determining whether the cross-correlation between daily median CT values and each epidemiological signal is statistically different from 0 (1-sample, two-tailed *t* test). CT: cycle threshold; MSA: metropolitan statistical area; R_t_: transmission rate; TX: Texas.

A 32- to 34-day shift was observed between the median CT value and the daily count of hospitalized individuals with COVID-19 (Figure 3). Although the visual inspection of the daily median CT, daily R_t_, and percent positivity plots over time (Figure 1) suggested that peaks in R_t_ and percent positivity followed a trough in median CT, no statistically significant time delays were detected between the median CT value and change in percent positivity (*P*=.41) or R_t_ (*P*=.32). Pairwise comparisons revealed that several demographic factors of the testing samples were associated with COVID-19 outbreak measures (Table 2).

The data set in this study was substantially larger than those reported in comparator studies but differed in that it was not from a surveillance sample. Instead, this study used samples from individuals who required testing due to the presence of COVID-19 symptoms or required testing in the absence of symptoms (eg, for work or travel clearance). The median CT value was the most common measure of the population distribution of CT values across research studies to date, and R_t_ and percent positivity were the most common outbreak measures (Table 3).

**Table 1:** Demographic characteristics of the population from the greater El Paso area based on the COVID-19 tests submitted to the Dascena COVID-19 Laboratory between September 15, 2020, and January 11, 2021.

Characteristic		Value
Age (years), mean (SD)		36.92 (18.53)
**Gender, n (%)**		
	Female	81,520 (54.93)
	Male	66,270 (44.65)
	Prefer not to answer	390 (0.26)
	Unknown	230 (0.15)
**Ethnicity and race, n (%)**		
	Hispanic	127,722 (86.06)
	White (non-Hispanic)	6668 (4.49)
	Black or African American (non-Hispanic)	1891 (1.27)
	Asian or Pacific Islander (non-Hispanic)	879 (0.59)
	Native American or Alaskan (non-Hispanic)	317 (0.21)
	Other (non-Hispanic or prefer not to answer)^a^	10,933 (7.37)

^a^This category includes individuals who indicated “other” or “multiracial” (Black and White) for race or had no race and ethnicity data documented.

**Table 2:** Demographic factors of COVID-19 testing samples collected between September 15, 2020, and January 11, 2021, that were correlated with measures of COVID-19 outbreak dynamics during the sample collection period.

Measure of COVID-19 outbreak dynamics	Correlated variable	Correlation coefficient	*P* value
Daily R_t_^a^	Daily median age	−.332	<.001
Daily changes in percent positivity	Daily percentage of samples from Hispanic individuals	.265	.006
Daily COVID-19 hospitalization (33-day shift)	Daily median age	−.451	<.001

^a^R_t_: transmission rate.

**Table 3:** Comparison of studies that examine cycle threshold (CT) values of SARS-CoV-2–positive samples at the population level

Study characteristics	This study	Hay et al [24]	Walker et al [25]	El Zein et al [31]
Source population	El Paso, Texas metropolitan statistical area	Nearly all hospital admissions into Brigham & Women’s Hospital in Boston, Massachusetts	United Kingdom’s national COVID-19 Infection Survey, which provided a representative sample of the United Kingdom	All patients who tested positive for SARS-CoV-2 at the Detroit Medical Center
Type of testing	Testing based on symptoms or testing for nonsymptomatic purposes (eg, travel and work)	2 weeks of symptomatic testing and 4.5 months of surveillance testing	Surveillance testing	—^a^
Sample size	148,410 samples and 36,306 positive tests	—	843,85 samples and 1892 positive tests	708 hospitalized patients and 282 ambulatory patients
Biological sample types included	Mostly nasopharyngeal swabs and small numbers of anterior nares, salivary, or unknown sample types.	Nasopharyngeal swabs	Nose and throat swabs	Nasopharyngeal swabs
Timeframe (duration)	September 15, 2020, to January 11, 2021 (around 4 months)	April 3, 2020, to November 10, 2020 (around 7 months)	April 26, 2020, to October 11, 2020 (around 5.5 months)	April 4, 2020, to June 5, 2020 (around 2 months)
Gene target(s) of RT-PCR^b^	Nucleocapsid, spike, and *ORF1ab*^c^ genes	—	Nucleocapsid, spike, and *ORF1ab* genes	Nucleocapsid gene
CT-based value(s)	Daily median CT value	Distribution, median, and skew of CT values	Mean and median CT values	High, medium, and low viral load (CT values of ≤25, 26-36, and ≥37, respectively)
Outbreak measure	R_t_^d^, count of individuals hospitalized with COVID-19, and change in percent positivity	R_t_	Positivity rate	Mortality
Outcome(s) of study	Negative correlation between median CT and R_t_, negative correlation between median CT and hospitalization count (with time delay), and negative correlation between median CT and percent positivity	Correlation between R_t_ and median and skewness of CT values among positive surveillance specimens	Declines in mean and median CT values preceded increases in positivity rates.	Downward trend in viral load coincided with a decrease in the number of deaths

^a^Not available.

^b^RT-PCR: reverse transcription polymerase chain reaction.

^c^*ORF1ab*: open reading frames 1ab.

^d^R_t_: transmission rate.

## Discussion

### Principal Findings

In this study, we conducted an exploratory analysis of potential correlations between the population distribution of CT values for SARS-CoV-2–positive samples and COVID-19 dynamics. Our results show that the daily median CT value negatively correlated with three measures of COVID-19 dynamics, namely the daily SARS-CoV-2 R_t_, the daily count of COVID-19 hospitalizations (with a time delay), and the daily change in percent positivity for SARS-CoV-2 detection among testing samples in the greater El Paso area (Figure 2).

At present, pandemic surges are largely predicted based on observed local case and mortality rates, which may lag behind changes in transmission rates by several weeks or be obscured by changes in testing capacity [24]. Given the ubiquitous availability of CT data and the pressing nature of the pandemic, interest has risen in exploring the possibility that the population distributions of CT values can be used as indicators of local outbreaks. This study adds to the growing literature on this topic by providing an analysis of median CT values from samples collected from an entire geographical area and contextualizing the results via a comparison to other research investigating the application of population-based CT values.

In the greater El Paso area, daily median CT values were found to negatively correlate with the daily percent positivity rate among samples, the daily R_t_ values extracted from community case rates, and the daily count of COVID-19 hospitalizations (with a delay). Of note, these associations were not observed in supplementary analyses (Figures S1, S2, and S3 in Multimedia Appendix 1) conducted for different Texas MSAs where substantially fewer tests, which covered a smaller proportion of the population (Table S1 in Multimedia Appendix 1), were processed. There appeared to be great day-to-day variability in the median CT values over time rather than consistent trends in the MSAs evaluated in supplementary analyses. This potentially reflects differences in the strength of signals that could be detected. In addition, substantial differences in the study populations may have contributed to the variable significance of the relationship between median CT value and outbreak measures among study sites. This hypothesis is supported by the observation of significant demographic differences between the El Paso MSA and the Texas MSAs evaluated in the supplementary analyses (Table S2 in Multimedia Appendix 1). This observation indicates that certain qualities of data sets that are used to measure population CT values may be important to their utility in approximating local COVID-19 pandemic surges.

Changes in the population distribution of CT values significantly (*P*<.001) preceded a rise in COVID-19 hospitalization counts in El Paso. However, contrary to the a priori hypothesis that changes in CT values would precede pandemic surges, the cross-correlation plots of median CT values, percent positivity rates, and R_t_ values did not strongly demonstrate such a relationship. It therefore remains unclear from the data whether changes in the population distribution of CT values preceded changes in community transmission or vice versa. Other studies evaluating population CT values of surveillance samples have reported that changes in CT values may precede traditional signs of an outbreak [24,25]. The inclusion of tests that were based on the presence of symptoms in the sample population may have influenced this association, such that a decline in CT values may be more closely linked to current case rates.

### Strengths

The strengths of this study include the fact that all RT-PCR analyses were conducted at a single laboratory that used standardized testing protocols and that large samples of positive COVID-19 tests were acquired for the study site. The vast majority (147,720/148,410, 99.54%) of samples were nasopharyngeal swabs. As such, differences in median CT values based on sample type likely did not impact results. This study was not limited to a single medical center but included samples collected from an entire geographical area. This study compared median CT values to R_t_ values and hospitalization counts—traditional public health benchmarks that are used to define pandemic surges—thereby providing greater validity than what would be possible with only an internal comparison of different metrics for testing sample data. In addition, this study provided a novel examination of the features of RT-PCR testing data, which may contribute to and affect the usability of population-level metrics of CT values in predicting disease dynamics in a community.

### Limitations

Although the study sample was large, other variables and forms of bias (eg, sampling bias) may have influenced the results. Indeed, differences in the comprehensiveness of the El Paso data set versus those in the supplementary site data sets (ie, the relative proportion of tests conducted by the Dascena laboratory versus those of other testing providers) may have contributed to the skew in the supplementary samples. Future directions for research on population-based CT values may therefore include analyzing whether significant differences in results can be detected in different subsamples of tested populations and evaluating methods for collating CT data across testing providers in a given geographic area.

No data on symptomatology were associated with samples at the time of collection. As such, these data did not allow us to distinguish between samples collected as part of a clinical evaluation of symptoms consistent with COVID-19 or those collected for other reasons (eg, clearance for work or travel). Prior research assessing the population distribution of CT values in relation to community outbreaks has explicitly used surveillance samples [24,25]. The variability in the observed correlations between the median CT value and outbreak measures in El Paso or those in other testing locations may partially reflect variability in the proportions of tests that were based on the presence of symptoms and tests for nonsymptomatic purposes in a given location. However, other differences between the testing site populations may also have contributed to the observed variability in the relationship between the median CT value and outbreak measures, such as differences in the demographics of the tested population. The research question of whether median CT values derived from all testing data, instead of those derived from surveillance-only testing data, may be reliably used to predict disease outbreaks remains unresolved and can only be addressed by using data sets in which symptomatology at the time of testing or reasons for testing may be linked to test results.

The samples used in this study were not collected expressly for the purposes of public health surveillance or research; therefore, the demographic composition of the sampled population varied from day to day. As indicated by Table 2, some aspects of the daily demographic composition of the tested population were found to correlate with epidemiological outcomes. Daily variability in the sampled population may therefore translate to variability in the strength of the associations between median CT values and measures of disease dynamics. However, these associations may also reflect underlying epidemiological trends, such as the disproportionately high rates of COVID-19 infection among Hispanic individuals [33], including those that occur during outbreaks. Additional research with real-world samples may build on this study by further exploring the relevance of demographic factors to the accuracy and utility of population-based CT measures.

### Conclusions

As national, state, and local authorities continue to refine public health programs to track and contain the spread of SARS-CoV-2, it is imperative to optimize methods for predicting surges in community transmission. Greater lookahead times would enable local and state officials to enact public health policies for mitigating an anticipated pandemic surge and would provide health systems with the opportunity to initiate changes to their standard operating procedures, including activating reserve clinical personnel, procuring additional resources to the extent possible, and converting facilities to support additional patient flow. The population distribution of CT values, as measured by the median CT value, is a potential indicator for local outbreaks, which merits further investigation and may warrant the tracking of these quantitative data by public health departments.

## Appendix

Multimedia Appendix 1 Supplementary materials.

## Abbreviations

CT: cycle threshold
IRB: Institutional Review Board
MSA: metropolitan statistical area
*ORF1ab*: open reading frames 1ab
R_t_: transmission rate
RT-PCR: reverse transcription polymerase chain reaction
