# Supplementary material for: Correlation of Population SARS-CoV-2 Cycle Threshold Values to Local Disease Dynamics: Exploratory Observational Study
Source: JMIR Public Health Surveill. 2021 Jun 3;7(6):e28265. doi: 10.2196/28265 (PMC8176948; doi:10.2196/28265)
Supplement: Multimedia Appendix 1 [file publichealth_v7i6e28265_app1.docx]

**Supplementary Material**

**(A)**

**
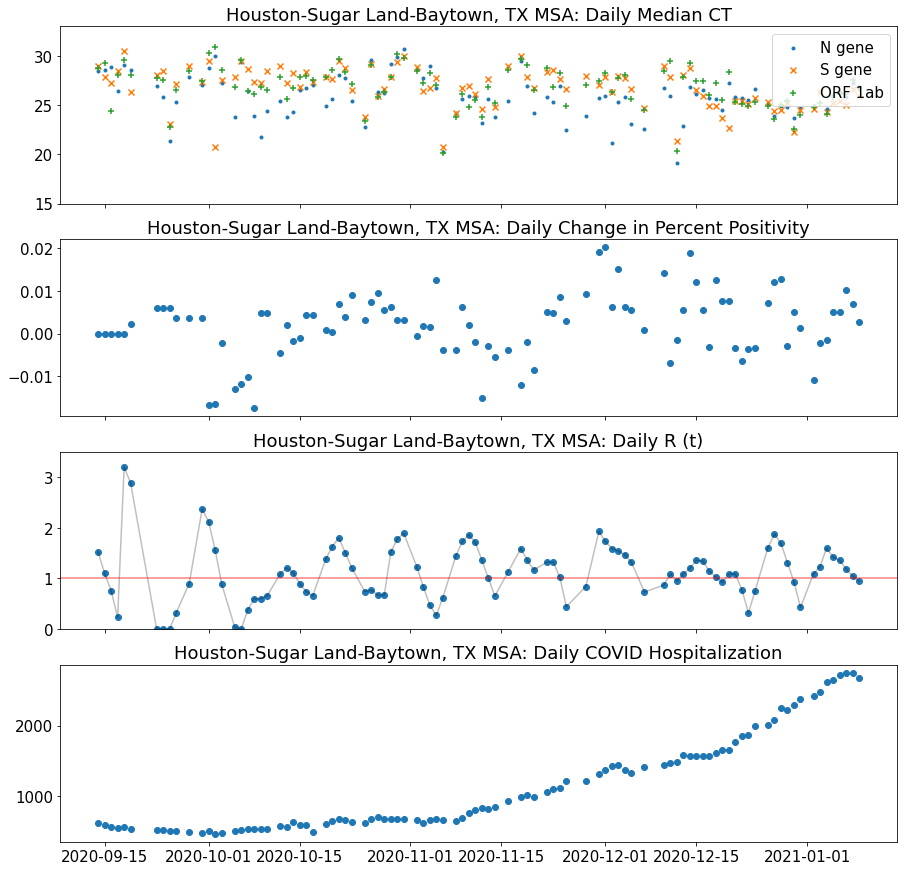

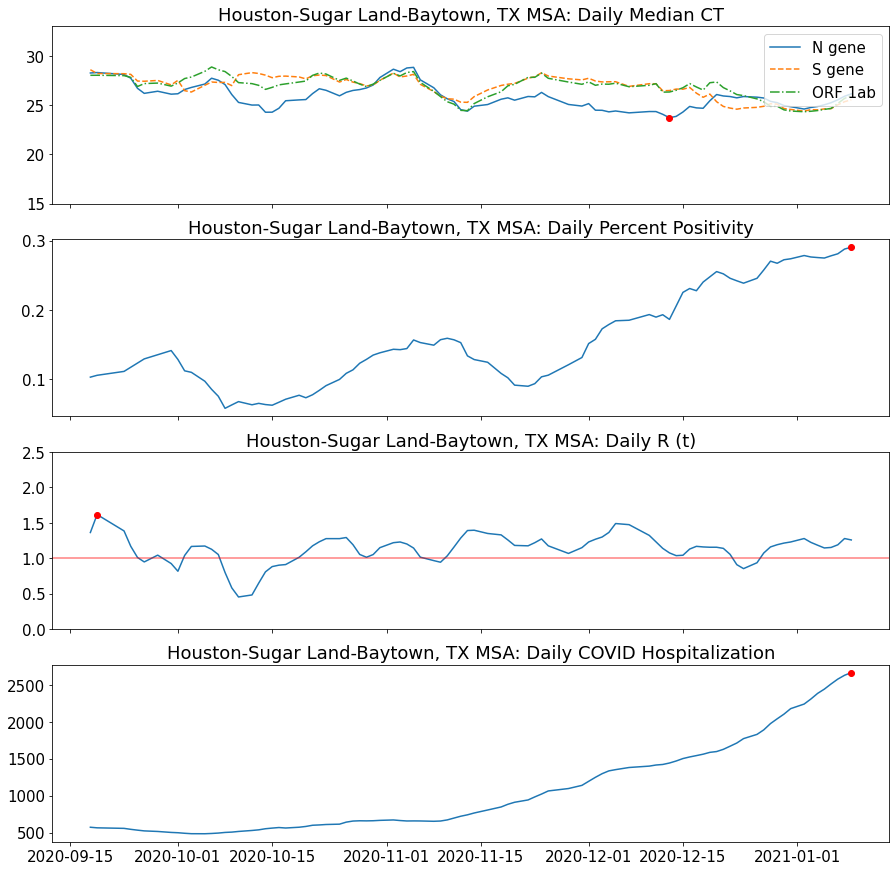
**

**(B)**

**
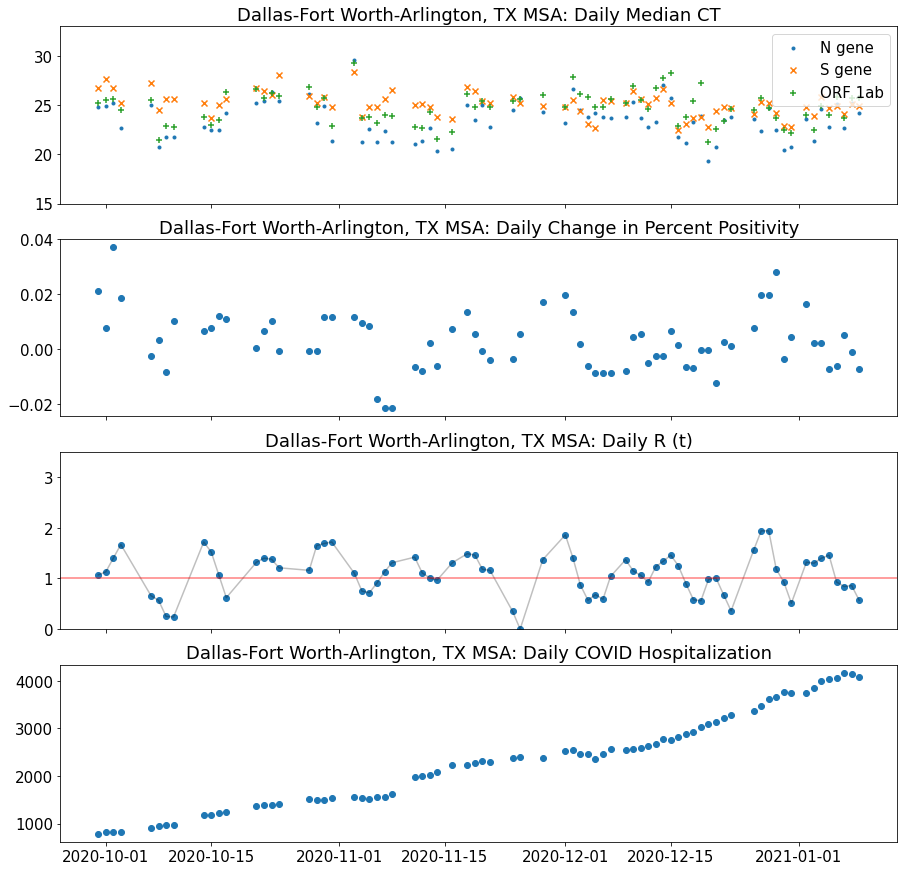

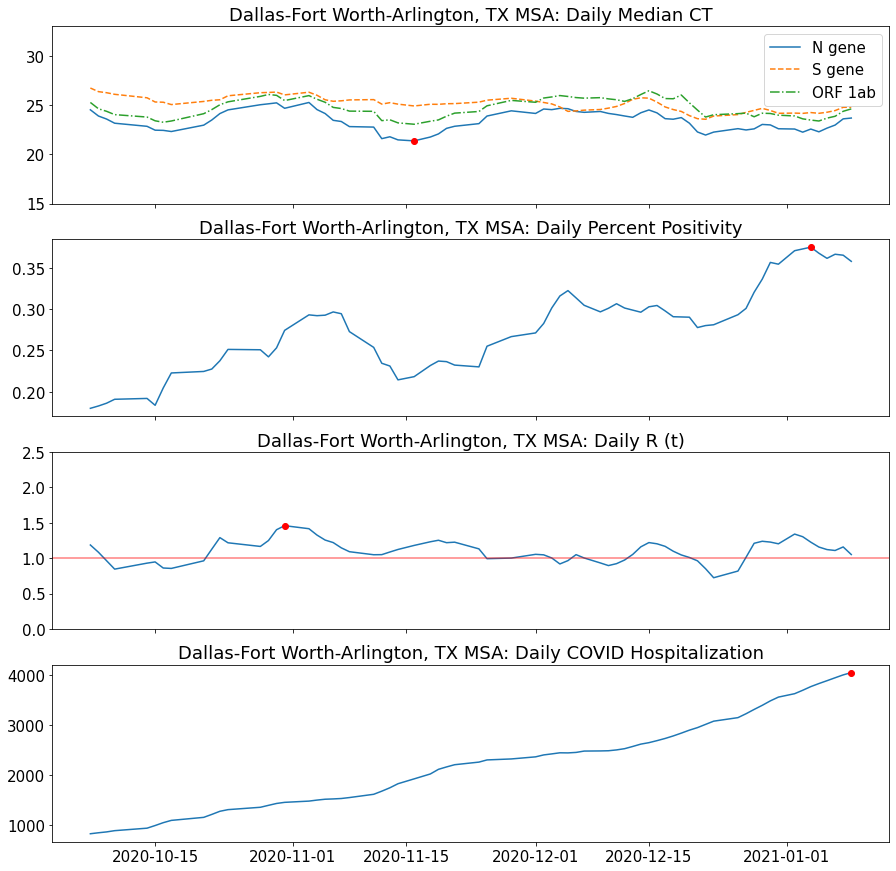
**

**(C)**

**
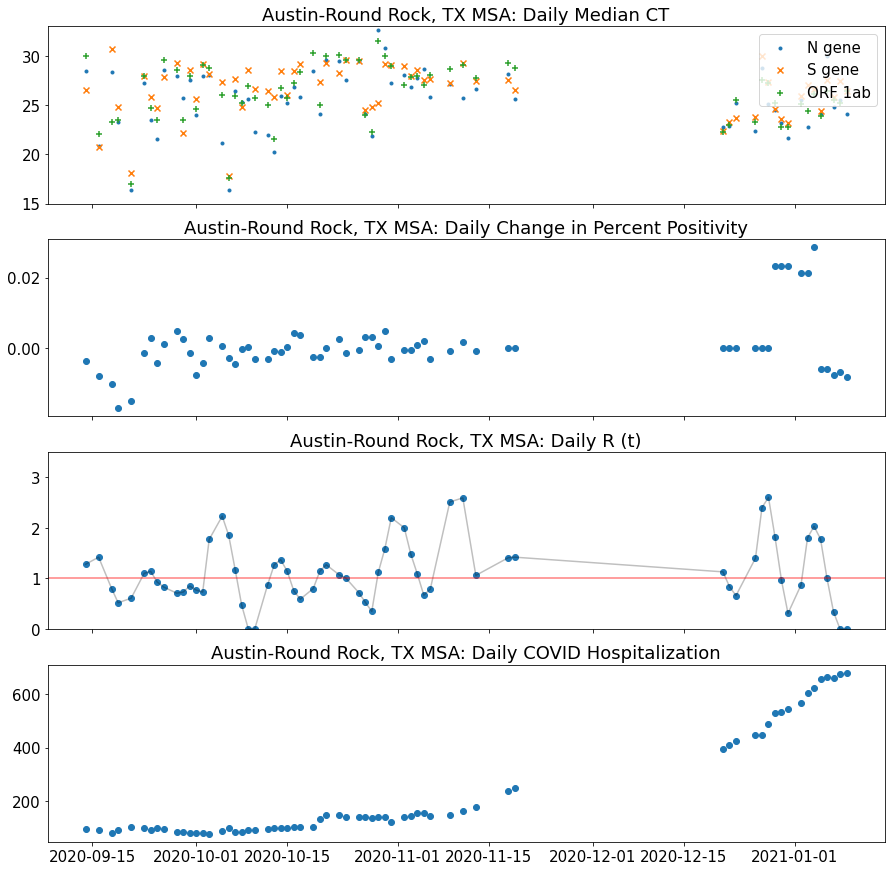

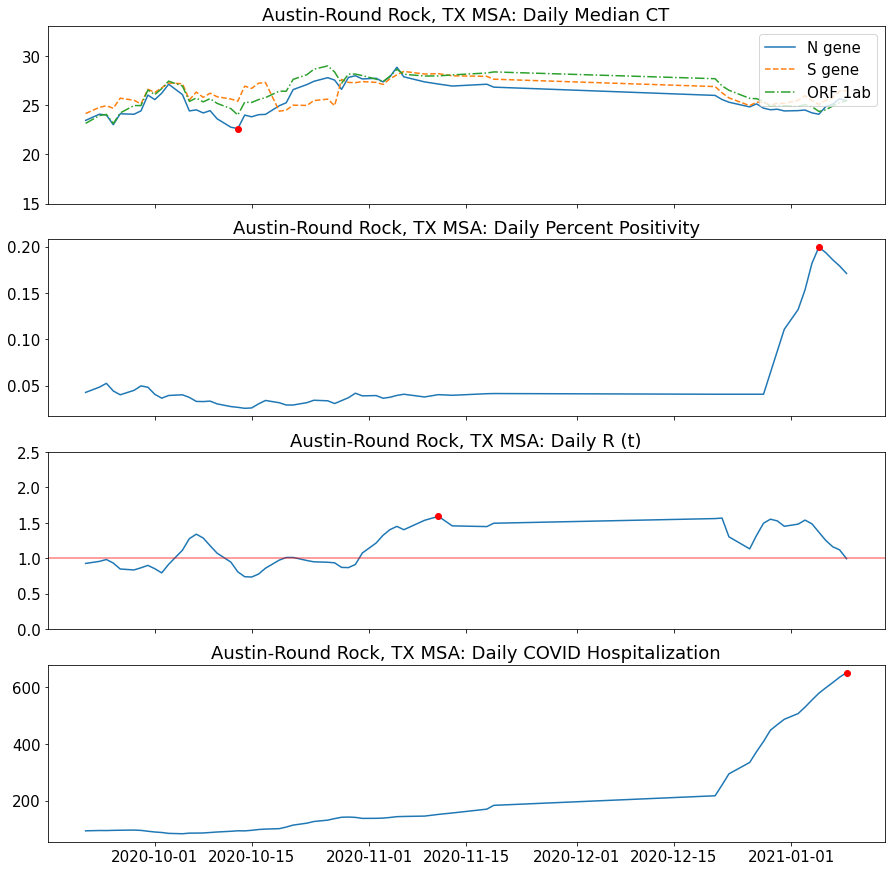
**

**Supplementary Figure 1.** Daily median cycle threshold (CT) value for SARS-CoV-2 positive samples, daily SARS-CoV-2 transmission rate R(t), daily number of individuals hospitalized with COVID-19, daily change in percent positivity of SARS-CoV-2 detection, 7-day rolling average of the daily median CT value for SARS-CoV-2 positive samples, 7-day rolling average of daily SARS-CoV-2 R(t), 7-day rolling average of the number of individuals hospitalized with COVID-19 and rolling average of the percent positivity of SARS-CoV-2 detection among Dascena samples in (A) Houston-Sugarland-Baytown Metropolitan Statistical Area (MSA), (B) Dallas-Fort Worth-Arlington MSA, and (C) Austin-Round Rock MSA between September 15th, 2020 and January 11th, 2021

**(A)**

**
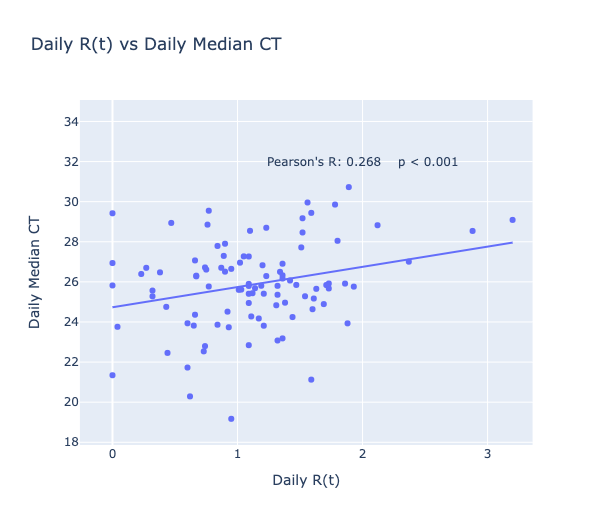

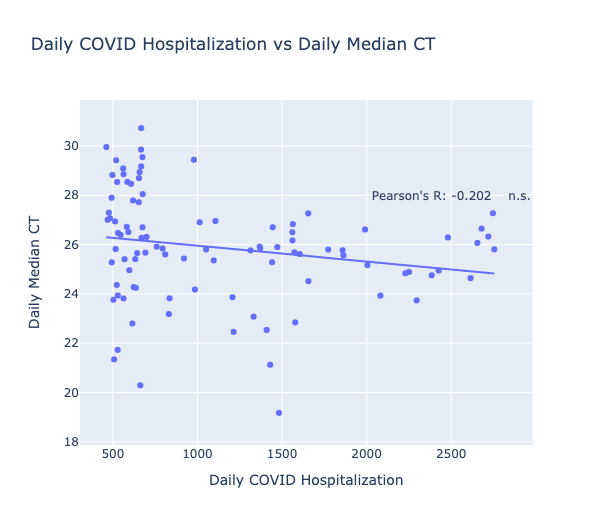

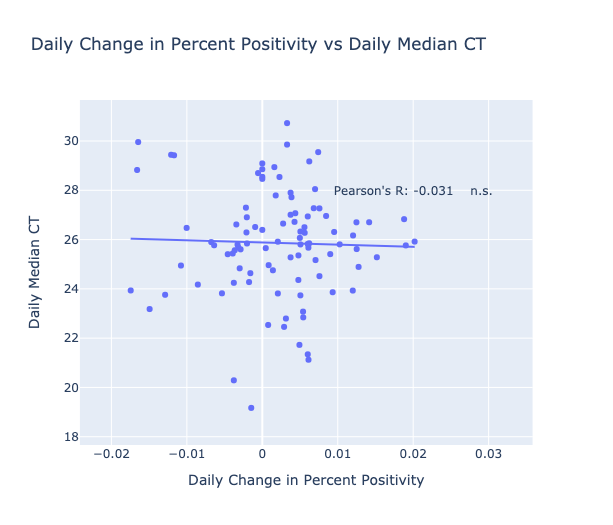
**

**(B)**

**
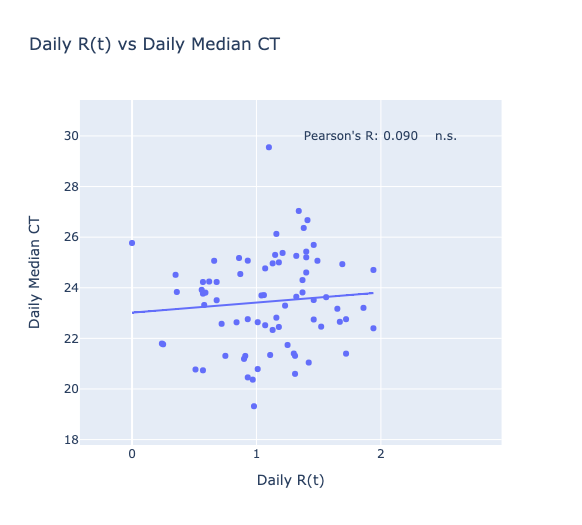

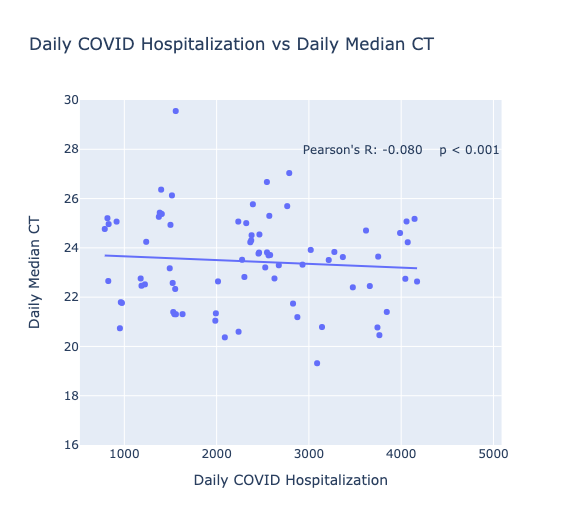

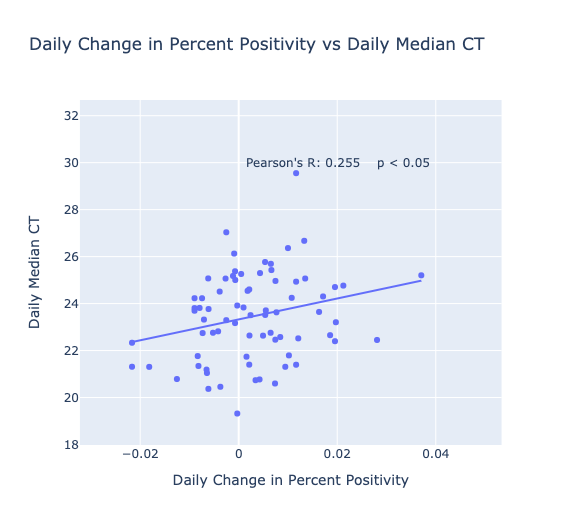
**

**(C)**

**
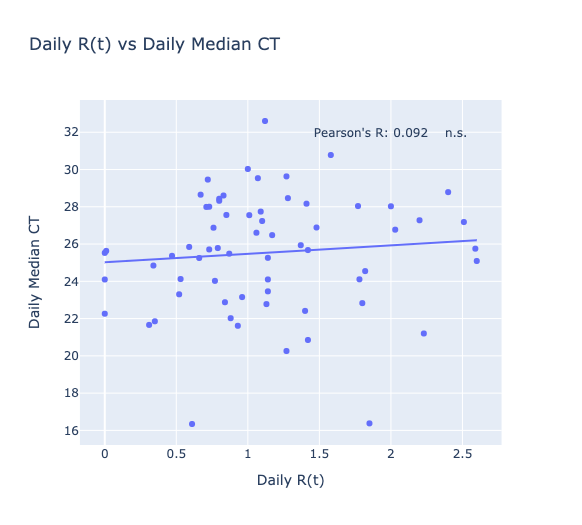

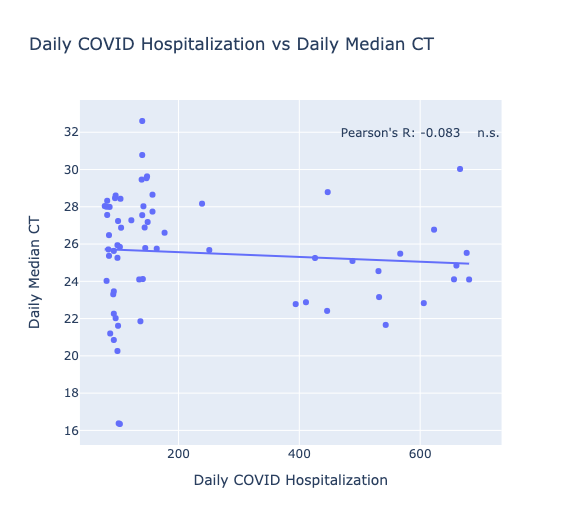

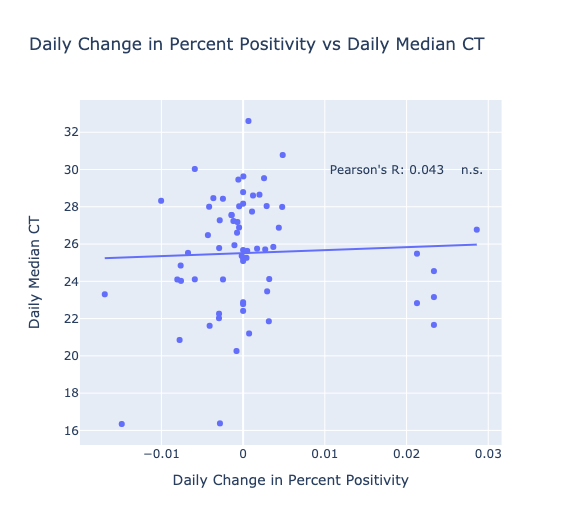
**

**Supplementary Figure 2.** Linear regression and scatterplots of daily median cycle threshold (CT) values of SARS-CoV-2 positive samples versus daily SARS-CoV-2 transmission rate R(t), daily number of individuals hospitalized with COVID-19, and daily change in percent positivity for SARS-CoV-2 detection in (A) Houston-Sugarland-Baytown Metropolitan Statistical Area (MSA), (B) Dallas-Fort Worth-Arlington MSA, and (C) Austin-Round Rock MSA between September 15th, 2020 and January 11th, 2021

**(A)**

**
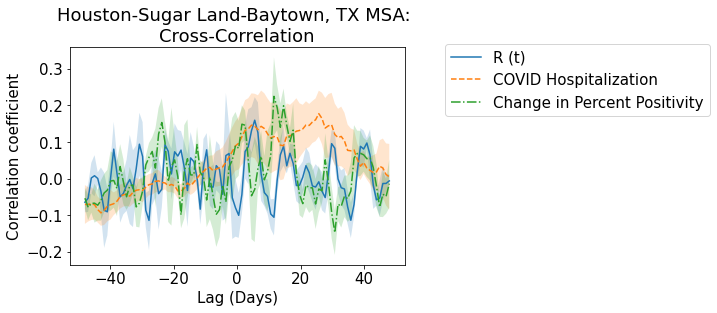
**

**(B)**

**
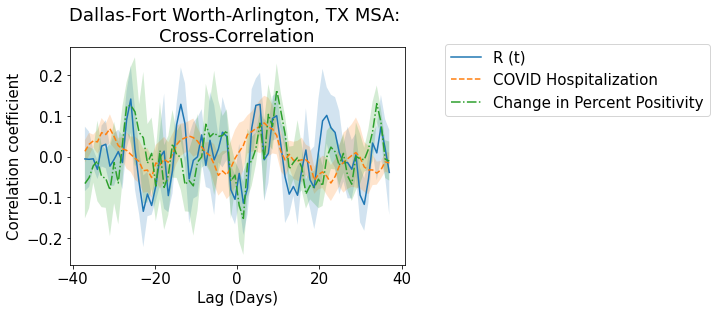
**

**(C)**

**
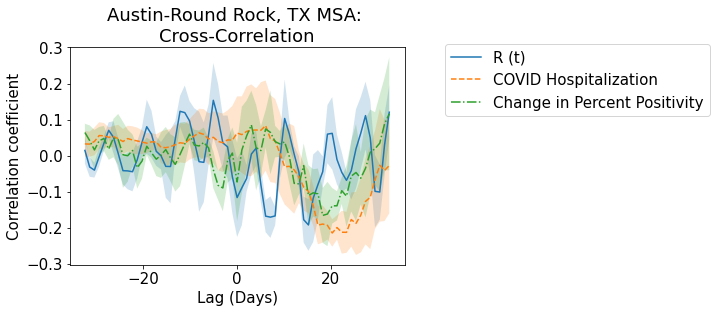
**

**Supplementary Figure 3.** Cross-correlation plots of daily median cycle threshold (CT) values of SARS-CoV-2 positive samples versus daily SARS-CoV-2 transmission rate R(t), daily number of individuals hospitalized with COVID-19, and daily change in percent positivity for SARS-CoV-2 detection in (A) Houston-Sugarland-Baytown Metropolitan Statistical Area (MSA), (B) Dallas-Fort Worth-Arlington MSA, and (C) Austin-Round Rock MSA between September 15th, 2020 and January 11th, 2021

**Supplementary Table 1.** Volume of COVID-19 tests per population in additional testing sites, compared to El Paso Metropolitan Statistical Area (MSA) between September 15th, 2020 and January 11th, 2021

| **Metropolitan statistical area (MSA)** | **Population** | **Number of tests conducted during study period** | **Tests per 100,000 individuals** | **Number of positive tests conducted during study period** | **Positive tests per 100,000 individuals** |
| --- | --- | --- | --- | --- | --- |
| El Paso | 844,124 | 148,410 | 17,582 | 36,306 | 4,301 |
| Houston-Sugar Land-Baytown | 7,066,141 | 45,625 | 646 | 9,276 | 131 |
| Dallas-Fort Worth-Arlington | 7,573,136 | 34,279 | 453 | 9,539 | 126 |
| Austin-Round Rock | 2,227,083 | 16,730 | 751 | 1,177 | 53 |

**Supplementary Table 2.** Demographics for all COVID-19 test samples sent to the Dascena COVID-19 Laboratory (between September 15th, 2020 and January 11th, 2021) from each additional Metropolitan Statistical Area (MSA), compared to the El Paso MSA using a z-test with a significance level of p<.05. * p<.01 ** p <.001

|  | **El Paso** | **Houston-**  **Sugarland-**  **Baytown** | **Dallas-**  **Fort Worth-**  **Arlington** | **Austin-**  **Round Rock** |
| --- | --- | --- | --- | --- |
| **Age (Mean, SD)** | 36.92 (18.53) | 37.48 (19.19) ** | 39.12 (19.23) ** | 35.78 (15.13) ** |
| **Gender** | | | | |
| **Female** | 81,520 (54.93%) | 25,369 (55.60%) | 19,564 (57.07%) ** | 9,022 (53.93%) |
| **Male** | 66,270 (44.65%) | 19,870 (43.55%) ** | 14,584 (42.54%) ** | 7,396 (44.21%) NS |
| **Prefer Not to Answer** | 390 (0.26%) | 13 (0.03%) ** | 69 (0.20%) * | 2 (0.01%) ** |
| **Unknown** | 230 (0.15%) | 370 (0.81%) ** | 62 (0.18%) | 310 (1.85%) ** |
| **Race** | | | | |
| **Hispanic** | 127,722 (86.06%) | 20,648 (45.26%)** | 17,198 (50.17%)** | 4,907 (29.33%)** |
| **White, non-Hispanic** | 6,668 (4.49%) | 4,947 (10.84%)** | 8,596 (25.08%)** | 7,996 (47.79%)** |
| **Black or African American, non-Hisanic** | 1,891 (1.27%) | 6,495 (14.24%)** | 4,262 (12.43%)** | 966 (5.77%)** |
| **Asian or Pacific Islander, Non-Hispanic** | 879 (.59%) | 1,790 (3.92%)** | 1,145 (3.34%)** | 1,494 (8.93%)** |
| **Native American/Alaskan, Non-Hispanic** | 317 (.21%) | 133 (.29%)** | 154 (.45%)** | 52 (.31%)** |
| **Other/Prefer not to Answer, Non-Hispanic** | 10,933 (7.37%) | 11,603 (25.43%)** | 2,923 (8.53%)* | 1,314 (7.85%) |
